# Melatonin as an Antitumor Agent against Liver Cancer: An Updated Systematic Review

**DOI:** 10.3390/antiox10010103

**Published:** 2021-01-12

**Authors:** Paula Fernández-Palanca, Carolina Méndez-Blanco, Flavia Fondevila, María J. Tuñón, Russel J. Reiter, José L. Mauriz, Javier González-Gallego

**Affiliations:** 1 Institute of Biomedicine (IBIOMED), University of León, Campus of Vegazana s/n, 24071 León, Spain; pferp@unileon.es (P.F.-P.); cmenb@unileon.es (C.M.-B.); ffonp@unileon.es (F.F.); mjtung@unileon.es (M.J.T.); jgonga@unileon.es (J.G.-G.); 2 Centro de Investigación Biomédica en Red de Enfermedades Hepáticas y Digestivas (CIBERehd), Instituto de Salud Carlos III, Av. de Monforte de Lemos, 5, 28029 Madrid, Spain; 3 Department of Cell Systems & Anatomy, UT Health San Antonio Long School of Medicine, San Antonio, TX 78229, USA; reiter@uthscsa.edu

**Keywords:** cholangiocarcinoma, hepatocellular carcinoma, liver cancer, melatonin

## Abstract

Melatonin (*N*-acetyl-5-methoxytryptamine) is an indoleamine with antioxidant, chronobiotic and anti-inflammatory properties; reduced levels of this hormone are associated with higher risk of cancer. Several beneficial effects of melatonin have been described in a broad number of tumors, including liver cancers. In this work we systematically reviewed the publications of the last 15 years that assessed the underlying mechanisms of melatonin activities against liver cancers, and its role as coadjuvant in the treatment of these tumors. Literature research was performed employing PubMed, Scopus and Web of Science (WOS) databases and, after screening, 51 articles were included. Results from the selected studies denoted the useful actions of melatonin in preventing carcinogenesis and as a promising treatment option for the primary liver tumors hepatocellular carcinoma (HCC) and cholangiocarcinoma (CCA), either alone or in combination with other compounds. Different processes were modulated by the indole, such as inhibition of oxidative stress, proliferation, angiogenesis and invasion, promotion of immune system response, cell cycle arrest and apoptosis, as well as recovery of circadian rhythms and autophagy modulation. Taken together, the present systematic review highlights the evidence that document the potential role of melatonin in improving the landscape of liver tumor treatment.

## 1. Introduction

Primary liver cancer constitutes the sixth most prevalent type of tumor and is the fourth common cause of cancer-related mortality worldwide [1]. A total of 841,080 new cases were diagnosed in 2018, with an estimated 781,631 deaths and an age-standardized mortality rate of 8.5/100,000. The most frequent types of primary liver cancer in adults are hepatocellular carcinoma (HCC) and cholangiocarcinoma (CCA), constituting HCC the 70–85% of cases and CCA the 30–15%, depending on the country [2]. Liver cancer also appears in children and adolescents, accounting hepatoblastoma (HB) and HCC for 67–80% and 20–33% of cases, respectively [2,3].

Unfortunately, most liver cancer patients are diagnosed at advanced stages when surgical treatment is no longer an available option [4]. Moreover, the high mortality associated with liver cancer is related to its lack of sensitivity and development of resistance to a few treatments that lead to the chemotherapy failure. These drawbacks may be explained, at least in part, by the various phenotypes and histological characteristics of tumor liver cells and their microenvironment [5]. Furthermore, the high refractoriness of liver cancer to treatments has been associated with the interaction of very complex and diverse mechanisms of chemoresistance, which can act synergistically to protect tumor cells from the chemotherapy agents [3].

Melatonin (*N*-acetyl-5-methoxytryptamine), the main product of the pineal gland, is an indoleamine with antioxidant, chronobiotic and anti-inflammatory properties [6]. A reduction of melatonin levels or a depressed excretion of its main metabolite, 6-sulfatoxymelatonin, have been related to an increased cancer risk, suggesting an anticancer role of this indoleamine [7]. Collectively, the published data strongly support the oncostatic actions of melatonin on different types of tumors, including liver cancer [8,9,10].

The inhibitory role of melatonin in hepatic tumors involves a number of different molecular and cellular processes including reduction of cellular proliferation, cell cycle arrest, limiting angiogenesis and metastasis and promoting cell death [10,11,12,13,14,15,16,17]. Moreover, melatonin reportedly increases the sensitivity of liver cancer cells to currently available treatments [4,18].

In the present article, we systematically review the scientific literature published in the last 15 years which is focused on the main molecular and cellular mechanisms associated with the melatonin’s antitumor effects in liver cancers, and its potential usefulness in improving treatment of these tumor types.

## 2. Materials and Methods

### 2.1. Search Strategy

This systematic review has been conducted followed the Preferred Reporting Items for Systematic Reviews and Meta-Analyses (PRISMA) guidelines [19] (Table S1). For comprehensive literature searches, we conducted the article search in PubMed, Scopus and Web of Science (WOS) from inception to 9 October 2020, identifying a total of 369 articles, 88 from PubMed, 148 from Scopus and 133 from WOS.

The full search strategy was the following for each database:

1. PubMed: (“melatonin” [All Fields]) AND (“liver cancer” [All Fields] OR “liver tumor” [All Fields] OR “hepatocarcinoma”[All Fields] OR “HCC”[All Fields] OR “hepatocellular carcinoma”[All Fields] OR “angiosarcoma”[All Fields] OR “cholangiocarcinoma”[All Fields] OR “hepatoblastoma”[All Fields]).
2. Scopus: TITLE-ABS-KEY ((“melatonin”) AND (“liver cancer” OR “liver tumor” OR “hepatocarcinoma” OR “HCC” OR “hepatocellular carcinoma” OR “angiosarcoma” OR “cholangiocarcinoma” OR “hepatoblastoma”)).
3. WOS: TS = ((“melatonin”) AND (“liver cancer” OR “liver tumor” OR “hepatocarcinoma” OR “HCC” OR “hepatocellular carcinoma” OR “angiosarcoma” OR “cholangiocarcinoma” OR “hepatoblastoma”)).

### 2.2. Inclusion and Exclusion Criteria

We applied the following inclusion criteria to select articles: (1) employment of liver cancer as model of study; (2) use of melatonin as treatment either as individual or in combination with another agent; (3) articles that reported antioxidant or antitumor effects of melatonin. Articles that met the following criteria were excluded: (1) review articles or similar compilations; (2) conference or congress communications; (3) full-text articles in a language other than English; (4) studies published prior to 2005.

### 2.3. Study Selection

Two authors independently conducted the study selection and any disagreement was solved by a third author. After performing full search of original articles, duplicates among databases were removed and articles were subjected to screening. Papers were then selected based on the inclusion criteria and they were individually assessed, discarding those that met exclusion criteria. Finally, the original papers that met the all eligibility criteria were identified and included in the qualitative analysis.

### 2.4. Data Extraction

Data from included articles were extracted by two independent authors, compiling in a table the following information: name of first author and publication year, country, type of liver tumor, experimental model (in vitro or in vivo) and sample size, melatonin administration strategy, treatment regimen (dose, frequency and duration) and alterations in the studied processes.

## 3. Results and Discussion

### 3.1. Study Selection and Characteristics

**Figure 1:**
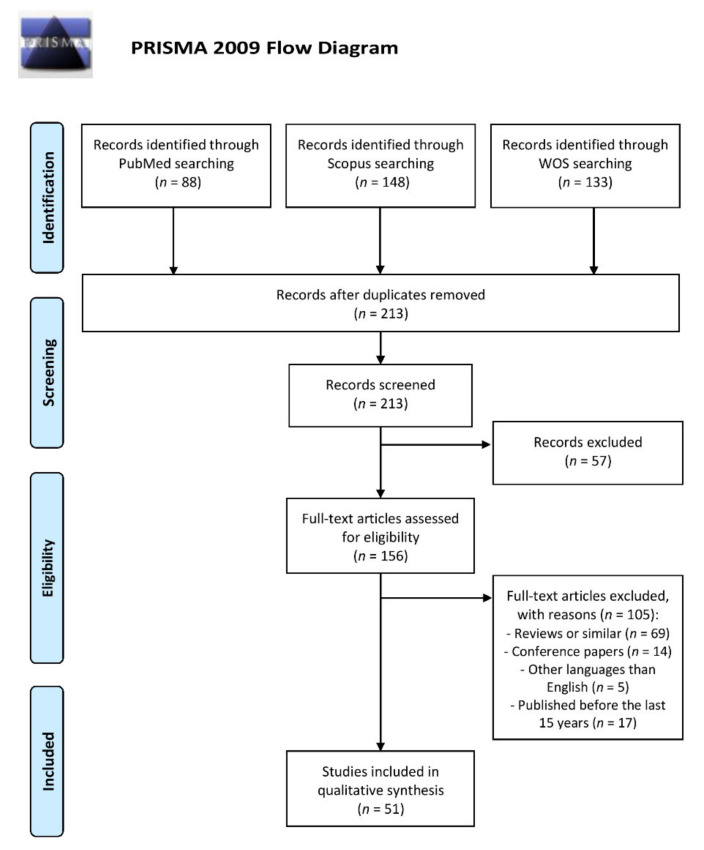
Flow diagram of the search and study selection performed according to the Preferred Reporting Items for Systematic Reviews and Meta-Analyses (PRISMA) statement. WOS, Web Of Science.

After the database searches and identification of original articles, 156 duplicates were removed, and 213 articles were screened by title and abstract. Among them, 57 papers did not meet the inclusion criteria and, thus they were discarded. The remaining 156 articles were individually analyzed and 105 of them which met exclusion criteria were then removed, leaving 51 publications identified as relevant studies that were included in this systematic review. The process of study selection is described in Figure 1.

**Figure 2:**
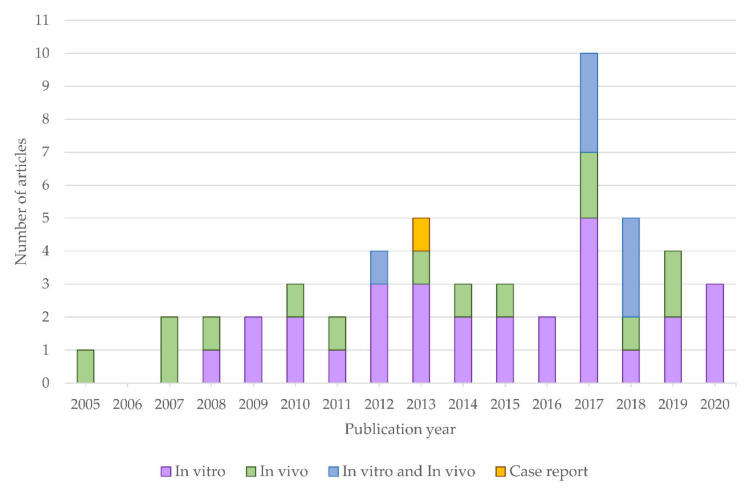
Temporal distribution of the number of articles published after 2004 which evaluate melatonin effects in liver tumors with only in vitro or in vivo models, with both type of models or in a case report.

An increasing number of articles reporting beneficial properties of melatonin in liver tumors were published in the last years (Figure 2). The main data provided by the included studies are compiled in Table 1. Among these publications, five studies were performed with CCA models, and the remaining 46 used HCC models, which is in accordance with the previously mentioned fact that HCC and CCA are the main primary liver tumors. It is also noted that 50 articles analyzed the properties of melatonin as single treatment, while 32 evaluated potential combinations with chemotherapeutic agents, specific inhibitors or anticancer compounds. Regarding the experimental model, 21 studies investigated melatonin effects employing animal models, 17 in HCC and four in CCA, while 36 articles involved in vitro analysis, 35 in HCC and only one in CCA, finding also one case report in HCC. Although most articles used similar cellular or animal models (human HepG2 cell line and DEN-induced liver injury for HCC; KKU-M055 and KKU-M214 cells lines and *Opisthorchis viverrini* administration for CCA), the range of melatonin concentration was wide, from 0.01 nM to 10 mM in cell culture, and from 4 to 50 mg/kg in animal experiments.

In recent years, there has been a steady increase the number of publications using both in vivo and in vitro methods carried out to analyze the role of melatonin in reducing hepatocarcinogenesis. This was the case for studies that used melatonin as a sole treatment or when it was used in association with other molecules.

This following section summarizes the main findings obtained within the 15 last years. Since HCC constitutes the most frequent liver cancer, the majority of the data are related to this cancer phenotype; findings on other cancer types is summarized when available.

### 3.2. Melatonin as Antioxidant and Chronobiotic Agent in Liver Cancer

Carcinogenetic processes often involve oxidative stress, and administration of antioxidant molecules may reduce the damage to cancerous hepatocytes [61,62,63]. Melatonin acts as an indirect antioxidant and as a direct free radical scavenger and displays an important role as an immunomodulatory and chronobiotic agent in different tissues, including liver [6,14,64,65].

In an animal HCC model induced by diethylnitrosamine (DEN) administration to rats, increases in liver transaminases (aspartate and alanine transaminase, AST and ALT respectively), α-fetoprotein, liver weight, and tumor incidence (which are indexes of tumor liver damage) were detected. Moreover, hepatocarcinogenesis induced lipid peroxidation (measured as thiobarbituric acid reactive substances—TBARS), and decreased serum activity of superoxide dismutase (SOD), catalase (CAT), glutathione peroxidase (GPx), glutathione S-transferase (GST), and reduced glutathione (GSH), which is a major antioxidant system in all tissues. Melatonin administration (5 mg/kg body weight for 20 weeks) abrogated these changes and reduced oxidative stress and hepatocarcinogenesis [22]. In other experiments using this same in vivo model of HCC, authors administered additionally CCl_4_ with DEN during tumor induction, founding increased AST and ALT; but, curiously, in this situation TBARS and GSH levels, as well as GPx and GST activities were also augmented, which may be due to the greater tumor cell proliferation. Melatonin administration (5 mg/kg body weight for 20 weeks) reversed all these changes to near control levels, which likely contributed to its hepatoprotective role [20].

Despite the known antioxidant action of melatonin, under certain circumstances this indoleamine can induce an increase in oxidative stress. Our group has demonstrated that melatonin (1 and 2 mM) co-administration with 2.5 mM sorafenib (a multikinase inhibitor drug that constitutes the standard HCC treatment), in Hep3B cells derived from human HCC, produced reactive oxygen species (ROS) accumulation into mitochondria. Further, this effect induced mitochondrial membrane depolarization and subsequently activation of cell death by mitophagy, a process that may be useful to improve tumor cell sensitivity to sorafenib [18].

Unfortunately, information related to the antitumor effects of melatonin involving its antioxidant ability in other liver cancers is more limited. Some experiments carried out using human CCA cell lines (KKU-M055 and KKU-M214) have demonstrated that melatonin administration (0.5, 1 and 2 mM for 48 h) increases ROS and cytochrome C production leading to cell death by apoptosis in a concentration-dependent manner [39]. Moreover, it has been found in an in vivo model of CCA in hamsters developed by infection with the trematode *Opisthorchis viverrini* that melatonin administration at different doses (5, 10 and 20 mg/kg body weight for 30 days) reduces in a dose-dependent manner ALT activity and the oxidative/nitrosative stress. Additionally, melatonin lowered the mRNA expression of oxidant generating genes such as inducible nitric oxide synthase (iNOS), nuclear factor-kappa B (NF-κB), cyclooxygenase-2 (COX-2) and the proinflammatory cytokines tumor necrosis factor alpha (TNF-α) and interleukin 1 beta (IL-1β). These effects were accompanied by an increase in the expression of genes encoding antioxidant proteins including nuclear erythroid 2-related factor 2 (Nrf2) and manganese superoxide dismutase (Mn-SOD), a reduction of liver injury (assessed by heme oxygenase activity) and a diminution of cytokeratin-19 (CK-19) expression—indicating a minor bile duct cell proliferation [26]. These results suggest that oxidative and nitrosative stress modulation by melatonin could be useful not only against HCC but also in CAA treatment and prevention.

Since circadian clock plays a key role in liver physiology and chronodisruption is known to augment hepatocarcinogenesis [66], different experiments have been carried out to analyze the association between the antioxidant and chronobiotic effects of melatonin in HCC. Thus, a preliminary study performed in an HCC model induced by DEN in Indian male field mice (*Mus booduga*) compared melatonin (0.5 mg/kg body weight) and oxaliplatin (2 mg/kg body weight) effects when administered for 10 weeks; the endpoints included evidence of oxidative stress and circadian locomotor rhythm perturbations. Results showed that both drugs ameliorated the changes in AST and ALT activities induced by DEN, and normalized SOD, CAT, GPx and GST antioxidant activities without any significant differences between melatonin and oxaliplatin-treated animals. However, melatonin was more effective than oxaliplatin in counteracting the modifications induced by DEN in the circadian locomotor activity rhythms [38].

These results have been confirmed in another study, using a similar model of HCC induced by DEN in rats, in which melatonin (5 mg/kg body weight for 20 weeks) and α-ketoglutarate (2 g/kg body weight for 20 weeks) antitumor effects associated with changes in the circadian rhythms of the antioxidant system were compared. In this study, authors observed that liver detectable tumors were fully prevented by melatonin or α-ketoglutarate. Moreover, DEN administration increased significantly mesor and amplitude of circadian rhythms of AST and ALT activities and plasma α-fetoprotein (with delay of AST and ALT acrophase, and α-fetoprotein phase advance); melatonin or α-ketoglutarate administration partially counteracted these effects. Furthermore, DEN induced changes in the mesor values of different antioxidant parameters (TBARS mesor increase, but reduction of GSH concentration mesor and SOD, CAT, GPx and GST activities), where acrophases were delayed in all cases. Melatonin, but not α-ketoglutarate, reduced TBARS mesor and augmented SOD mesor; however, changes in acrophases were only partially prevented by administration of melatonin or α-ketoglutarate. Collectively, these results suggest that melatonin, similar to other HCC antitumor molecules such as α-ketoglutarate, can modulate 24-h-rhythms of lipid peroxidation and antioxidants [23].

The results of other studies are in concordance with the previous findings. Thus, it has been shown that melatonin modulates the circadian rhythm (acrophase, amplitude, and mesor) of lipid peroxidation, antioxidant enzymes and GSH levels. In vivo experiments, inducing HCC by DEN administration in rats, have found that hepatocarcinogenesis induced higher mesor values of lipid peroxidation (measured as TBARS production), increments in the acrophase of SOD, CAT, GPx and GSH, together with reductions in mesor values of these antioxidant enzymes and GSH. These alterations in the circadian rhythms may be due to enhanced utilization of these antioxidants during hepatocarcinogenesis and were reversed by melatonin administration (5 mg/kg body weight for 20 weeks), suggesting that its antitumor effects could be associated, at least in part, with its dual antioxidant and chronobiotic dual ability [21].

Finally, circadian rhythms modulation by melatonin has been implicated not only with regulation of antioxidant enzymes but also with some metabolic changes. An in vitro and in vivo combined study, using 7288CTC HCC cells and 7288CTC tumor xenografts in situ, has shown that exposure to daytime blue-enriched LED light can reduce tumor progression by a mechanism associated with increase in the endogenous melatonin levels. This drives the inhibition of hepatoma metabolism through suppression of the Warburg effect (glucose uptake and lactate production) and impeding cyclic AMP (cAMP)-depending linoleic acid uptake [52].

### 3.3. Cell Cycle Arrest by Melatonin in Liver Cancer

Carcinogenesis is a multistage process usually promoted by a disturbed and uncontrolled cell proliferation, with cell cycle dysregulation, associated with an increase in tumor cell viability. In HCC, 1 mM melatonin exposure of HepG2 cells induce a drop in percentage of cells in the G0/G1 phase, and an increase of cells in the S phase, confirming the antiproliferative effect of the methoxyindole [24]. In a more extensive study, our group demonstrated that in vitro administration of melatonin (0.1–10 mM for 10 days) causes a dose- and time-dependent reduction of HepG2 human HCC cell viability; these results were associated with modifications in the cell cycle kinetics, with an increase in the percentage of cells in the G0/G1 phase accompanied by a reduction in cell counts in G2/M phase; these changes were related to the induction of p53 expression and subsequent activation of p21 (a potent inhibitor of cell cycle kinases—CDKs) [10]. Results were recently confirmed by other authors, not only in HepG2 but also in Hep3B HCC cells; they reported a reduction of cyclin D1 expression and the induction of cell cycle arrest by 1 and 2 mM melatonin [59].

Increases in the protein expression of the main melatonin receptors in liver (MT1, MT3, and retinoic acid-related orphan receptor alpha—RORα), which could be driving the cytostatic and antiproliferative effects of this indole, have been found in HepG2 cells after 1 and 2.5 mM melatonin administration [11]. MT1 activation seems to be related to modulation of cAMP and members of mitogen-activated protein kinases (MAPKs) family such as c-Jun N-terminal kinase 1 (JNK), extracellular signal-regulated kinase (ERK) and p38, the deregulations of which are often found in different types of cancer including HCC [12]. Furthermore, in CCA, in vitro and in vivo experiments and data from human biopsies have demonstrated a reduction on endogenous melatonin levels but increases in MT1 and MT2 receptors, which could constitute a compensatory mechanism by this tumor to retard the progression of cell growth induced by a diminished melatonin endogenous secretion [28].

Curiously, in vitro experiments using HepG2 HCC cells exposed to magnetic field (50 Hz, 10 µT), which acts as an alleged cell proliferation inducer, reported that 10 nM melatonin administration produces cytostatic and antiproliferative effects. This change was accompanied by a reduction in proliferating cell nuclear antigen (PCNA) expression, a nuclear nonhistone protein necessary for DNA synthesis which is usually elevated during G1/S phase [30].

Other studies, using an in vivo model of HCC induced by DEN in mice, have observed that melatonin administration (5 and 10 mg/kg body weight, daily for 10, 20, 30 and 40 weeks) induces both p21 and p53 expression in a time- and dose-dependent manner; the indole also alleviates the expression of proliferative/cell cycle regulatory proteins (Ki67, PCNA, cyclin D1, cyclin E, CDK4, and CDK6), reducing the activity of transcription factors such as NF-κB and signal transducer and activator of transcription 3 (STAT-3), and modulating the sphingosine kinase/sphingosine 1-phosphate (SphK/S1P) system. All mentioned elements are frequently associated with hepatocarcinogenesis [47]. Moreover, in in vitro studies using Hep3B HCC cells, it has been demonstrated that these effects are associated, at least in part, with normalized expression of the brain and muscle arnt-like protein 1 (Bmal1) and circadian locomotor output cycles protein kaput (Clock) proteins —which constitute components of the circadian machinery in hepatocarcinogenesis —when 0.5 and 1 mM melatonin was administered [14].

Combined melatonin administration can improve the cytostatic and antiproliferative effects of other molecules against HCC such as sorafenib. Indeed, the combination of 0.2–2 mM melatonin and sorafenib enhanced the cell cycle arrest at G0/G1 phase in both Bel-7402 and SMMC-7721 HCC cell lines by upregulating p27—an inhibitor of several CDKs, and downregulating *p*-AKT and also different proteins including c-myc, cyclin D1 and CDK4/6, which play key roles in cell proliferation. These results were validated using an in vivo model of xenograft tumor treated with 25 mg/kg body weight (daily for 18 days) [46]. More recently, a preliminary study using HepG2 HCC cells, has also confirmed that 3 mM melatonin increased the antiproliferative effects of (–)-epigallocatechin-3-gallate (EGCG) through a p21-associated mechanism [56].

### 3.4. Modulation of Apoptosis by Melatonin in Liver Cancer

Resistance to apoptosis is one of the main hallmarks of cancer and its selective induction in cancer cells has emerged as an interesting possibility for new antitumor treatments. In liver, apoptotic signaling is transduced mainly via two molecular pathways: the extrinsic (or death receptor-mediated) and the intrinsic (or mitochondria-dependent). The extrinsic pathway is initiated by death ligands, such as Fas-ligand (FasL), leading to cleavage of procaspase-8 to its active form, which subsequently upregulates downstream effectors such as caspase-3. Conversely, the intrinsic pathway is triggered by mitochondria dysfunction, resulting in changes in proteins of Bcl-2 family such as the mitochondrial translocation of the proapoptotic Bax protein, which can be mediated by members of the MAPK superfamily such as JNK and p38, cytochrome C release and activation of downstream effector caspases [67,68].

In vitro experiments using human HepG2 cells, have demonstrated that administration of melatonin (0.1–10 mM for 10 days) is able to induce both extrinsic and intrinsic pathways of apoptosis, with induction of FasL-independent caspase-8 activity, cytosolic cytochrome C release from mitochondria with upregulation of Bax, and induction of caspase-9 activity in a dose- and time-dependent manner. These mechanisms are responsible for induction of caspase-3 activity and poly(ADP-ribose) polymerase (PARP) proteolysis, leading to tumor hepatocyte death by apoptosis [10]. In these conditions, Bax activation is associated with Bim overexpression through the nuclear translocation and activation of the transcription factor Forkhead box protein O3 (FoxO3a) when exposed to 1 and 2 mM melatonin [29]. Additionally, in vitro studies using Hep3B HCC cells have demonstrated that modulation of genes implicated in the circadian machinery (such as Bmal1, Clock or Sirtuin 1—Sirt1) by 0.5 and 1 mM melatonin doses may be associated with increases in the Bax/Bcl-2 ratio and subsequently apoptosis induction [14].

Melatonin also sensitizes HCC cells to endoplasmic reticulum (ER) stress, thereby inducing apoptosis. In the study in question, 1 nM melatonin decreased COX-2 levels, which are usually elevated in liver tumor cells, promoting the apoptosis rate by elevating the levels of the proapoptotic transcription factor CHOP (GADD153), and reducing the ratio between the Bcl-2 antiapoptotic protein and the proapoptotic Bax (Bcl-2/Bax) in HepG2 cells [32]. Similar effects have been found by other authors, who additionally indicated that inhibition of COX-2 can be associated with changes in the activating transcription factor 6 (ATF-6) by 1 nM melatonin [41]. These results have been confirmed using an in vivo model of HCC induced by DEN in rats, where melatonin (1 mg/kg body weight, daily for 45 and 90 days) diminished COX-2 expression, which leads to the increased activity of CHOP and ATF-6, and reduced the Bcl-2/Bax ratio, causing an apoptosis response through upregulation of the activity of caspases-3, -8 and -9, and PARP proteolysis [17].

Information related to the proapoptotic effect of melatonin alone in other liver cancers is more limited. However, as indicated above, experiments performed using human CCA cell lines (KKU-M055 and KKU-M214) have demonstrated that melatonin concentrations of 0.5, 1 and 2 mM for 48 h increases ROS and cytochrome C production, leading to augmentation of caspase-3 and caspase-6 expression and subsequent apoptosis in a dose-dependent way [39]. These results have been confirmed in an in vivo model of CCA in hamsters, induced by DEN and co-infection with *Opisthorchis viverrini*. In this study, melatonin administration (50 mg/kg body weight, daily for 120 days) activated the intrinsic pathway of apoptosis through reduction on Bcl-2/Bax ratio, increment of cytochrome C release and subsequent caspase-3 activation. This was caused by a mechanism associated with alleviation on oxidative and nitrosative DNA damage which is related to the activation of Nrf2 and inhibition of NF-κB pathways [35]. Contrary results were found by Colombo et al. 2018 [51], where 1 mM melatonin induced NF-κB protein expression in HepG2 cells [51].

Various studies have indicated that melatonin sensitizes tumor liver cells to apoptosis when co-administered in combination with doxorubicin, a highly effective anticancer drug with a broad spectrum of activity. In this study, 10 nM melatonin in combination with doxorubicin initiates a synergistic proapoptotic effect in HepG2 and Bel-7402 HCC cells through the intrinsic pathway of apoptosis by upregulation of Bax, downregulation of Bcl-2 and activation of caspase-3 [25]. Ranges from 1 nM to 1 mM of melatonin, by elevation of CHOP levels and reduction of expression of different apoptosis inhibitor proteins (survivin, cellular and X-linked inhibitor apoptotic proteins—cIAP and XIAP, respectively), reversed the ER stress-induced resistance to doxorubicin and increased apoptosis in HepG2 and SMMC-7721 HCC cell lines, through a COX-2/PI3K/AKT dependent mechanism which improved the cytotoxic response to this molecule [33,34].

Sorafenib, as indicated previously, is a first-line drug for the treatment of advanced HCC, but despite its capacity to increase survival in HCC patients, its success is quite low in the long-term owing to the development of resistant cells through several mechanisms [69]. 1–5 mM melatonin co-administration promotes sorafenib-induced apoptosis through synergistic activation of JNK/c-jun pathway and caspase-3 in a dose-dependent manner in HuH7 HCC cells [44]. Moreover, as indicated above, 50 µM melatonin also increased regorafenib-induced apoptosis; this molecule inhibits multiple kinases and has been approved for patients with sorafenib-resistant HCC [70]. It has been noted that melatonin can enhance the regorafenib-associated Sirt3 upregulation, promoting mitochondrial dysfunction with mitochondrial depolarization and induction of apoptosis in SMMC-7721 HCC cells [60].

Melatonin also increases the proapoptotic effect of cisplatin, an antitumor molecule frequently used in cancer treatment. In vitro experiments using Bel-7402, SNU-449, HepG2 and Hep3B HCC cells have demonstrated that combined treatment of cisplatin with 1 mM melatonin increases the intrinsic pathway of apoptosis and inhibits the NF-κB/COX-2 pathways, improving the therapeutic efficiency of cisplatin in HCC [43]. More recently, it has been also shown that, in HepG2 and Hep3B HCC cells, 1 and 2 mM melatonin heightens cisplatin-induced apoptosis by downregulating different antiapoptotic proteins (including Bcl-2, Bcl-xL or Mcl-1), and upregulating cleaved caspase-3 and PARP, effects apparently related to activation of Hippo signaling pathway, which promotes apoptosis and suppress cell proliferation [59].

Transcriptomic analysis of genes associated with 5-fluorouracil (5-FU) resistance, which is a routine chemotherapeutic agent, has revealed that a 2.5 mM melatonin dose induces FoxO signaling pathway in HepG2 and HuH7 cells and in samples from HCC patients, improving 5-FU sensitivity [58].

In recent years, there has been an increasing interest on the use of flavonoids as anticancer agents [62]. In vitro experiments have also demonstrated that 2 and 4 mM melatonin co-administration potentiates the proapoptotic effects of EGCG in HepG2 cells by inhibition of Bcl-2 and COX-2, resulting in an enhanced cytotoxicity against HCC cells [56].

Finally, in an in vivo model of HCC following DEN administration in rats, melatonin (20 mg/kg body weight, twice a week for 5 weeks) improved mesenchymal stem cells (MSCs)-based therapies against HCC through different pathways, including apoptosis by upregulation of Bax and caspase-3 and downregulation of Bcl-2 and survivin [54]. These results have been also reported by other authors in a similar model employing the same indole doses, showing that combination of MSCs and melatonin induces a greater activation of caspases-3 and -9 and subsequent apoptosis [55].

### 3.5. Modulation of Autophagy by Melatonin in Liver Cancer

Autophagy constitutes a bulk degradation system which plays a key role in the cellular homeostasis in order to promote adaptation and cell survival; however, when excessive stimulation leads to programmed cell death instead of survival in a number of different pathophysiological situations including tumorigenesis [71]. Therefore, in cancer cells autophagy can act as a double-edged sword removing newly malignant cells and damaged mitochondria at the early stages but inducing survival under hypoxia and ischemia conditions in the later phases, it can be also associated with chemotherapy resistance and tumor progression [18].

Some in vivo experiments have demonstrated that melatonin (10 and 20 mg/kg body weight, daily for 14 days) triggers an autophagy process in H22 tumor-bearing mice, with induction of Beclin-1 expression and increase on the conversion of microtubule-associated protein 1 light chain 3 (LC3)-I to LC3-II (protein associated with the autophagosome membrane) by reducing mammalian target of rapamycin (mTOR) and AKT signaling. Interestingly, blockage of this autophagic process by Beclin-1 RNAi or 3-methyladenine (3-MA) administration enhanced melatonin-associated apoptosis in H22 mouse HCC cells in vitro [31].

Ceramides have emerged as key effectors in autophagy modulation, mediating the crosstalk with apoptosis [72]. Our group has found that 2 mM melatonin administration could induce transiently autophagy in HepG2 cells associated to JNK phosphorylation, increasing Beclin-1 expression, p62 degradation, and co-localization of LC3-II with lysosomal associated membrane protein 2 (LAMP-2). Moreover, under these conditions, siRNA silencing of the autophagy related protein 5 (ATG5) suppressed the changes previously observed in LC3-II and p62 melatonin-induced, but sensitized HepG2 cells to melatonin-associated apoptosis (with augmentation on PARP proteolysis and caspase-3 activation). Furthermore, the indole increased ceramide levels, but disruption of ceramide synthesis by inhibition of serine palmitoyl transferase (SPT) or acid sphingomyelinase (ASMase) enzymes resulted in different mechanisms, whereas ASMase inhibition partially protected HepG2 cells against melatonin while SPT inhibition significantly enhanced cell death [16].

Modulation of autophagy seems to be involved in tumor cell sensitivity and resistance to current treatments against HCC such as sorafenib [73]. Other in vitro HCC experiments using HepG2 and Bel-7402 cells interestingly reported that co-administration of a lower melatonin doses (10 µM) with sorafenib downregulated sorafenib-induced autophagy, showing a reduction in LC3-II/LC3-I ratio and an augmentation of p62 expression, accompanied by an enhancement of apoptosis with increases in Bax expression and a decrement in Bcl-2 [45]. These results have been recently confirmed by other authors who performed experiments using both HCC cells (HepG2, 7721 and HuH7) and HCC specimens from patients. They reported that co-administration of low melatonin doses (10 µM) with sorafenib increases the tumor cells death, blocks the sorafenib-induced prosurvival autophagy through a reduction of Beclin-1, but overcoming apoptosis resistance. These results seem to have been associated with melatonin restrain of ER stress by a downregulation of both PKR-like ER stress kinase (PERK) and the activation transcription factor-4 (ATF-4), which in turn reduced Beclin-1 expression, leading to minor conversion of LC3-I to LC3-II. This blockage of the prosurvival autophagy may improve the antitumor sorafenib effects in HCC [57].

Mitophagy, a distinct form of autophagy, promotes turnover of damaged mitochondria entrapped in autophagolysosomes. As indicated above, melatonin (1 and 2 mM) co-administration with 2.5 mM sorafenib in Hep3B cells derived from human HCC, under normoxic conditions, produced ROS accumulation into mitochondria and mitochondrial membrane depolarization. After these changes, an early co-localization of mitochondria with lysosomes was detected, correlating with the expression of PTEN induced putative kinase 1 (PINK1) and Parkin, which are mitophagy markers. These effects potentiated tumor cell apoptosis (with increases on Bax expression and PARP hydrolysis) and were also associated with the reduction of tumor cell proliferation [18]. Conversely, under hypoxia conditions, which is a known phenotype associated with chemotherapy failure [74], mitophagy acted as a cytoprotective mechanism in HCC tumor cells. Thus, in Hep3B cells under hypoxia, it has been demonstrated that co-administration of 2 mM melatonin blocks the cytoprotective mitophagy induced by the hypoxic microenvironment after 5 mM sorafenib treatment; this blockage seems to be associated with the downregulation of the hypoxia inducible factor-1α (HIF-1α) through the inhibition of the mTOR complex 1 (mTORC1)/ribosomal protein S6 kinase beta-1 (p70S6K)/ribosomal protein S6 (RP-S6) pathway [4].

Co-administration of 1 mM melatonin with cisplatin, in HepG2 HCC cells, induced autophagy progression by increasing Beclin-1 and LC3-II, which was associated with the suppression of the overexpression of mTOR, as well as DNA excision repair cross complementary 1 protein (ERCC1) previously induced by cisplatin treatment. These results confirm that co-administration of melatonin benefits the antitumor effects of cisplatin in HCC [37].

### 3.6. Modulation of Angiogenesis and Invasion by Melatonin in Liver Cancer

Angiogenesis—the process of new blood-vessel growth from the existing vasculature—plays a key role facilitating tumor growth and the metastatic process, and results from a dynamic balance between proangiogenic and antiangiogenic factors. Angiogenesis has been widely related with progression, invasion and metastasis of liver tumors, and its inhibition represents a testable approach for the treatment of this type of cancer [75,76,77].

Normoxic conditions within the tumor microenvironment lead to HIF-1α ubiquitination and subsequent proteasomal degradation, whereas under hypoxia this factor stabilizes and translocates to the nucleus, inducing the expression of several genes including the proangiogenic vascular endothelial growth factor (VEGF) [69]. Moreover, under hypoxia, STAT-3 phosphorylation seems to enhance HIF-1α stabilization, forming a transcriptional complex together with CBP/p300 co-activator, thereby increasing VEGF expression and subsequent angiogenesis [78]. In vitro experiments, performed in HepG2 cells treated with 1 mM melatonin, have demonstrated that this molecule reduces the expression of VEGF and HIF-1α, impairing cell migration and invasion, and reducing cell proliferation under both normoxia and hypoxia conditions [40]. Additional in vitro experiments, also performed in HepG2 cells treated with 1 mM melatonin under CoCl_2_-induced hypoxia, showed that melatonin not only reduces cell proliferation but also interferes with the transcriptional activation of VEGF; this blocks angiogenesis and tumor cell invasiveness, through a reduction of physical interaction between phospho-STAT-3, HIF-1α, and CBP/p300 within the VEGF promoter [13].

Research carried out using HepG2 and HuH7 cells treated with 1 mM melatonin has demonstrated the antiproliferative effect of this indoleamine, accompanied by the inhibition of the cell migration and invasion capacities in a dose-dependent manner; these findings were also confirmed in an in vivo model of HCC xenograft transplantation where animals were treated with 40 mg/kg of body weight melatonin. Melatonin increased the forkhead box A2 (FOXA2) transcription factor expression, induced upregulation of long non-coding RNA carbamoyl-phosphate synthase 1 intronic transcript 1 (lncRNA-CPS1-IT1), which in turn blocked HIF-1α that suppressed epithelial-mesenchymal transition (EMT) progression, cell migration and invasion [48]. Decreases in progression and invasiveness in HCC have been also reported by suppression of DNA repair through the lncRNA RAD51 antisense RNA 1 (lncRNA RAD51-AS1) induction when melatonin was administered at concentrations of 1 mM in HepG2 cells [50]. Additionally, not only lncRNAs but also some micro-RNAs have been implicated in the anti-invasive and antimetastatic activities of melatonin in HCC. In the same way, also in HepG2 and HuH7 cell lines, administration of 1 and 2 mM melatonin induced the miRNA let7i-p activation, which in turn downregulated the ras activated factor 1 (RAF-1) proto-oncogene, impaired HCC cell migration [53].

Matrix metalloproteinases (MMPs) are a family of zinc-dependent endopeptidases which degrade extracellular matrix, and their overexpression contributes to HCC growth and metastasis by inducing angiogenesis [79]. Among these proteins, MMP-9 has been associated with several stages of tumor growth, vascular invasion, and metastasis [80]. It has been reported that 1 mM melatonin reduced IL-1β-induced MMP-9 activity, impairing cell invasion and motility in HepG2 cells. This effect was lined to the suppression of IL-1β-induced NF-κB translocation and transcriptional activity, leading to downregulation of MMP-9 gene expression, which was coincident with the upregulation of the MMP-9-specific inhibitor tissue inhibitor of metalloproteinases (TIMP)-1 by melatonin [15].

Some authors have indicated that HCC modulation by melatonin depends both on the characteristics of the tumor cells and on the concentration of the administered molecule. Indeed, an in vitro study using two different melatonin doses (1 µM and 100 µM) administered to two different HCC human cell lines, HCC24/KMUH which are not susceptible to amphotericin B (AmB)-induced stress and HCC38/KMUH which are susceptible, has produced conflicting results relative to the expression of proangiogenic and angiostatic chemokines under AmB-induced stress. Hence, expression of proangiogenic agents such as chemokine (C-C motif) ligand 2 (CCL-2) and interleukin (IL-8) was induced by both 1 µM and 100 µM melatonin administration in HCC24/KMUH cells, but curiously the expression of the angiostatic C-X-C motif chemokine ligand 10 (CXCL-10) was also increased. However, expression of CCL-2 and IL-8, together with another proangiogenic chemokine called C-X-C motif chemokine ligand 6 (CXCL-6) and the antioxidant enzyme Mn-SOD, were reduced by both melatonin concentrations in the HCC38/KMUH. This showed that under AmB stress conditions melatonin inhibits the proangiogenic signal and act as antioxidant substituting for the Mn-SOD function. The findings indicate that clinical application of melatonin in patients with HCC should consider the liver tumor characteristics for the optimization of indole concentrations [27].

### 3.7. Melatonin Immunomodulation in Liver Cancer

As indicated previously, melatonin functions as both an antioxidant and chronobiotic and also as an immunomodulatory agent [6,14,64,65]. Some case reports in humans have shown that 20 mg/daily melatonin co-administration together with immunotherapy consisting of interleukin-2 (IL-2) and Bacillus Calmette-Guerin, after the initial surgical removal of nodules, results in a reduction of HCC progression and development of new liver tumors [36]. However, additional studies analyzing the molecular and cell mechanisms implicated in melatonin’s immunomodulatory role on liver cancer are necessary.

Some relevant information has been obtained from an interesting in vitro and in vivo study using HCC-derived exosomes not treated (Exo-con) or treated with 0.1 mM melatonin-treated (Exo-MT), which were co-cultured with human THP-1 and mouse RAW264.7 monocyte/macrophage cell lines or injected into BALB/C nude mice. The outcomes of these experiments suggested that Exo-con upregulate the expression of programmed death ligand 1 (PD-L1) and the secretion of inflammatory cytokines (IL-6, IL-10, IL-1β and TNF-α) in macrophages, since these changes were blocked in Exo-Mt specimens STAT-3 inactivation, therefore modulating the immune response [42].

Similarly, in an in vivo model of CCA induced by DEN and co-infected with *Opisthorchis viverrine*, bile duct cell proliferation with induction on transforming growth factor β (TGF-β) expression was observed. These changes were accompanied by IL-17-producing T helper (Th17) cell infiltration and overexpression forkhead box P3 (foxp3)—which is a master regulator in the development and function of regulatory T cells, together with increases on inflammatory cells, especially eosinophils, and induction on NF-κB, COX-2 and IL-1 expression. Melatonin administration (50 mg/kg body weight, daily for 30 days) suppressed all these alterations, suggesting that the indole might be effective in CCA treatment and prevention by a dual mechanism implicating both immunomodulation and its anti-inflammatory capacity [49].

### 3.8. Limitations

To the best of our knowledge, this is the first systematic review that summarizes the antitumor effects of melatonin in the case of liver cancer and was written with the intent of providing a better understanding of the mechanisms involved. Nonetheless, there are some limitations, mainly due to the large amount and heterogeneity of the articles finally included. Although the antioxidant role of melatonin is well established, there are other investigations in which this indole promoted ROS generation both in HCC and CCA, which could be due to the large concentrations of melatonin sometimes used. This may be one of the main limitations; thus, in both in vitro but also in vivo the melatonin dose/concentration varied widely among experiments summarized herein. This hindered firm conclusions relative to the absolute efficacy of melatonin as a treatment for liver cancer. Fortunately, melatonin has not significant toxicity when given to animals including humans and can be given in amounts over a very large range. Furthermore, for years it has been known that the concentrations needed to document the oncostatic effects of melatonin in vitro are much higher than anticipated relative to in vivo findings obtained. This has been interpreted to mean that under in vitro conditions all the actions of melatonin cannot be manifested since the metabolism of cells in culture probably differ from those in vivo [81].

In terms of cell cycle, there is a lack of investigation on effects in CCA, while melatonin-induced in vivo cell cycle arrest is reported in all HCC studies. Similarly, assessment of autophagy in CCA is also limited, whereas it has been fully evaluated in HCC. Results from studies on autophagy are somewhat divergent, since autophagy acts as a double-edged process in cancer, and melatonin also exerts dual actions on the modulation of autophagy machinery depending on cellular microenvironment; context specificity relative to melatonin’s actions in cancer have also been previously reported [82]. Conversely, melatonin effects on angiogenesis and invasive and migratory abilities in HCC seem to be consistent among investigations, with some different results caused by different tumor characteristics and melatonin concentrations employed.

One of the least studied properties of melatonin in reference to liver cancer is immunomodulation. Although a clear immunomodulatory activity associated with the anti-inflammatory action of melatonin has been shown both in HCC and CCA, further studies are necessary to ascertain the underlying mechanisms. Although the number of studies in which combination strategies with melatonin were evaluated is limited, articles analyzing co-administration with antitumor drugs, flavonoids or inhibitor molecules demonstrate a potential role of melatonin in improving sensitivity of hepatic cancers to several drugs. In summary, there are still deficiencies in terms of the potential utility of melatonin as an anti-cancer agent, not only as a sole but also as combined treatment, on the different processes described herein.

## 4. Conclusions

**Figure 3:**
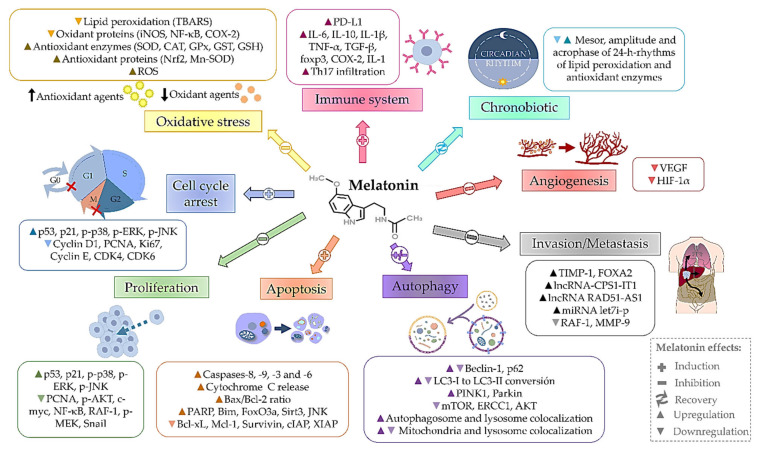
Cellular processes and protein expression modulated by melatonin in liver tumors. CAT, catalase; CDK, cyclin-dependent kinase; cIAP, cellular inhibitor apoptotic proteins; COX-2, cyclooxygenase-2; ERCC1, DNA excision repair cross complementary 1 protein; ERK, extracellular signal-regulated kinase; FOXA2, forkhead box A2; FoxO3a, forkhead box protein O3; foxp3, forkhead box P3; GPx, glutathione peroxidase; GSH, reduced glutathione; GST, glutathione S-transferase; HIF-1α, hypoxia-inducible factor 1α; IL-1, interleukin-1; IL-1β, interleukin 1 beta; IL-6, interleukin-6; IL-10, interleukin-10; iNOS, inducible nitric oxide synthase; JNK, c-Jun N-terminal kinase 1; LC3, microtubule-associated protein 1 light chain 3; lncRNA, long non-coding RNA; MEK, MAPK/ERK kinase 1; MMP-9, matrix metalloproteinase 9; Mn-SOD, manganese superoxide dismutase; mTOR, mammalian target of rapamycin; NF-κB, nuclear factor-kappa B; Nrf2, nuclear erythroid 2-related factor 2; PARP, poly(ADP-ribose) polymerase; PCNA, proliferating cell nuclear antigen; PD-L1, programmed death ligand 1; PINK1, PTEN induced putative kinase 1; RAF-1, ras activated factor 1; ROS, reactive oxygen species; Sirt3, sirtuin 3; Snail, zinc finger protein SNAI1; SOD, superoxide dismutase; TBARS, thiobarbituric acid reactive substances; TGF-β, transforming growth factor β; Th17, IL-17-producing T helper; TIMP-1, tissue inhibitor of metalloproteinases 1; TNF-α, tumor necrosis factor alpha; VEGF, vascular endothelial growth factor; XIAP, cellular and X-linked inhibitor apoptotic proteins.

This is the first systematic review compiling the antitumor effects exerted by melatonin in liver tumors, an unfortunately common and deadly cancer for which there are limited treatment options. Results from the studies reviewed here suggest that melatonin generally has beneficial properties in the prevention and treatment of the main primary liver tumors, HCC and CCA, by modulating a wide number of cellular processes, even protecting cells from the hepatotoxicity of other drugs. Among melatonin’s effects, as showed in Figure 3, it improves the immune response, enhances apoptosis, and positively influences the cell cycle and circadian rhythms; it also impairs tumor angiogenesis, invasion and cell proliferation. Considering its different roles on autophagy, melatonin may modulate this process depending on cellular context, always aimed at hindering tumor progression. Finally, combination studies with different molecule types, antitumor drugs, flavonoids and chemotherapeutics, provide evidence of the potential effects of melatonin as a coadjuvant agent to improve current treatments. Overall, this systematic review provides useful information and supports a role for melatonin as a promising drug in the treatment armamentarium of liver tumors.

## Figures and Tables

**Table 1:** Basic characteristics of experimental studies evaluating antitumor effects of melatonin against liver tumors.

**First Author,** **Publication Year,** **[Reference]**	**Country**	**Liver** **Tumor**	**Experimental Model (*n*, Sample Size per Group)**	**Administration Strategy**	**Treatment Regimen**	**Process Alteration**
Dakshayani et al. 2005 [20]	India	HCC	In vivo Adult male Wistar rats Intraperitoneal injection of DEN followed by subcutaneous CCl_4_ (*n* = 6)	Intraperitoneal melatonin	5 mg/kg 20 weeks	Antioxidant and hepatoprotective activity
Dakshayani et al. 2007 [21]	India	HCC	In vivo Adult male Wistar rats Intraperitoneal injection of DEN followed by subcutaneous CCl_4_ (*n* = 6)	Intraperitoneal melatonin	5 mg/kg Thrice a week 20 weeks	Chronobiotic effect Antioxidant effect
Subramanian et al. 2007 [22]	India	HCC	In vivo 3-months-old male Wistar rats Intraperitoneal injection of DEN followed by subcutaneous of CCl_4_ (*n* = 6)	Intraperitoneal melatonin	5 mg/kg Daily 20 weeks	Tumor growth inhibition Antioxidant activity
Martín-Renedo et al. 2008 [10]	Spain	HCC	In vitro Human HepG2 cell line	Melatonin	0.1–10 mM 4, 6, 8 and 10 days	Proliferation inhibition Apoptosis induction Cell cycle arrest
Subramanian et al. 2008 [23]	India	HCC	In vivo 3-months-old male Wistar rats Intraperitoneal injection of DEN followed by subcutaneous injection of CCl_4_ (*n* = 6)	Intraperitoneal melatonin	5 mg/kg Daily 20 weeks	Chronobiotic effect Antioxidant effect
Carbajo-Pescador et al. 2009 [11]	Spain	HCC	In vitro Human HepG2 cell line	Melatonin	1–2.5 mM 2, 4 and 6 days	Proliferation inhibition Cell cycle arrest
Ozdemir et al. 2009 [24]	Turkey	HCC	In vitro Human HepG2 cell line	Melatonin	0.05–1 mM 72 h	Cell cycle arrest
Fan et al. 2010 [25]	China	HCC	In vitro Human HepG2 and Bel-7402 cell lines	Melatonin + Doxorubicin	0.01–10 µM 48 h	Proliferation inhibition Apoptosis induction
Laothong et al. 2010 [26]	Thailand	CCA	In vivo Male Syrian golden hamsters Oral inoculation of 50 metacercariae of *Opisthorchis viverrini* (*n* = 5)	Oral melatonin	5, 10, 20 mg/kg Daily 30 days	Antioxidant and protective activity
Lin and Chuang 2010 [27]	Taiwan	HCC	In vitro Human HCC cell lines: HCC24/KMUH (resistant to AmB-induced oxidative stress) and HCC38/KMUH: (susceptible to AmB-induced oxidative stress)	Melatonin	1 and 100 µM 24 h	Proliferation increase
				Melatonin + AmB	1 and 100 µM 24 h	Antiangiogenic effect
Carbajo-Pescador et al. 2011 [12]	Spain	HCC	In vitro Human HepG2 cell line	Melatonin	1–2.5 mM 12, 24 and 48 h	Proliferation inhibition
Han et al. 2011 [28]	New York	CCA	In vivo 6-weeks-old male BALB/c nude mice Subcutaneous injection of Mz-ChA-1 cells (*n* = 4)	Intraperitoneal melatonin	4 mg/kg Thrice a week 43 days	Proliferation inhibition
Carbajo-Pescador et al. 2012 [29]	Spain	HCC	In vitro Human HepG2 cell line	Melatonin	50–2000 µM 1, 6, 24 and 48 h	Apoptosis induction
Cid et al. 2012 [30]	Spain	HCC	In vitro Human HepG2 cell line	Melatonin + MF exposure	0.01–1000 nM 4, 5 and 7 days	Proliferation inhibition
Liu et al. 2012 [31]	China	HCC	In vitro Mouse hepatoma cell line H22	Melatonin	100 µM 24 h	Apoptosis induction
				Melatonin + Beclin-1 RNAi Melatonin + 3-MA	100 µM 24 h	Autophagy blockade Apoptosis induction
			In vivo 8-weeks-old female BALB/c mice Subcutaneous injection of H22 cells (*n* = 10)	Intraperitoneal melatonin	10 or 20 mg/kg Daily 14 days	Autophagy induction
Zha et al. 2012 [32]	China	HCC	In vitro Human HCC HepG2 cell line Human hepatocyte HL-7702 cell line	Melatonin + Tunicamycin	10^−7^ µM 24 h	Proliferation inhibition Apoptosis induction
Carbajo-Pescador et al. 2013 [13]	Spain	HCC	In vitro Human HepG2 cell line	Melatonin	1 nM and 1 mM 2, 4, 6, 8, 12 and 24 h or 24 h	Hypoxia-dependent angiogenesis
Fan et al. 2013 [33]	China	HCC	In vitro Human HepG2 and SMMC-7721 cell lines	Melatonin + Doxorubicin	1 mM 24 h	Apoptosis induction
				Melatonin + Doxorubicin + Tunicamycin	0.1–1000 µM 24 h	Proliferation inhibition Apoptosis induction
Fan et al. 2013b [34]	China	HCC	In vitro Human HepG2 and SMMC-7721 cell lines	Melatonin	0.001–1000 µM 24 and 48 h	Proliferation inhibition Apoptosis induction
Laothong et al. 2013 [35]	Thailand	CCA	In vivo 4-to-6-weeks-old male Syrian golden hamsters Oral inoculation of 50 metacercariae of *Opisthorchis viverrini* and 12.5 ppm DEN (*n* = 15)	Oral melatonin	10 or 50 mg/kg Daily 120 days	Apoptosis induction
Tomov et al. 2013 [36]	Bulgaria	HCC	Case report 67-years-old female	Intermittent administration of IL-2, BCG and oral melatonin	20 mg Daily	Immunomodulation
Bennukul et al. 2014 [37]	Thailand	HCC	In vitro Human HepG2 cell line	Melatonin	0.5–5 mM 24 and 48 h	Autophagy induction
				Melatonin + Cisplatin		
Ordóñez et al. 2014 [15]	Spain	HCC	In vitro Human HepG2 cell line	Melatonin	1 mM 24 h	Angiogenesis and invasion inhibition
				Melatonin + IL-1β		
Verma et al. 2014 [38]	Malaysia	HCC	In vivo Adult male mice Intraperitoneal injection of DEN (*n* = 6)	Intraperitoneal melatonin	0.5 mg/kg Thrice a week 10 weeks	Antioxidant activity Modulation of circadian rhythms
Laothong et al. 2015 [39]	Thailand	CCA	In vitro Human KKU-M055 and KKU-M214 cell lines	Melatonin	0.5, 1 and 2 mM 48 h	Oxidative stress activity Apoptosis induction
Moreira et al. 2015 [17]	Brazil	HCC	In vivo Male Wistar rats Intraperitoneal injection of DEN and 2-AAF administration at week 4 (*n* = 12)	Oral melatonin	1 mg/kg Daily 45 and 90 days	Apoptosis induction
Ordóñez et al. 2015 [16]	Spain	HCC	In vitro Human HepG2 cell line	Melatonin	2 mM 0.5–48 h	Apoptosis induction Autophagy induction
Colombo et al. 2016 [40]	Brazil	HCC	In vitro Human HepG2 cell line	Melatonin	1–10^6^ nM 24 h	Proliferation inhibition
					1 mM 24 h	Inhibition of hypoxia-derived invasion
Prieto-Domínguez et al. 2016 [18]	Spain	HCC	In vitro Human HepG2, HuH7 and Hep3B cell lines	Melatonin	0.1–2 mM	Proliferation inhibition Pro-oxidant activity Mitophagy induction Apoptosis induction
				Melatonin + Sorafenib		
Bu et al. 2017 [41]	China	HCC	In vitro Human HepG2 cell line	Melatonin + Tunicamycin	10^−6^ –1 mM	Apoptosis induction and ER stress
Cheng et al. 2017 [42]	China	HCC	In vitro Human HepG2 and Bel-7402 cell lines	Melatonin	0.1 mM	Immunomodulation
			In vivo 6-weeks-old female BALB/c nude mice Injected with Exo-con or Exo-MT (0.1 mM melatonin)	Exo-MT	100 µL Daily 10 days	
Hao et al. 2017 [43]	China	HCC	In vitro Human Bel-7402, SNU-449, HepG2 and Hep3B 2.1-7 cell line	Melatonin	1 mM 48 h	Proliferation inhibition Inhibition of cell migration ability Apoptosis induction
				Melatonin + CDDP		
Lin et al. 2017 [44]	China	HCC	In vitro Human HuH7 cell line	Melatonin	1–5 mM 48 h	Proliferation inhibition Apoptosis induction
				Melatonin + Sorafenib		
Liu et al. 2017 [45]	China	HCC	In vitro Human HepG2 and Bel-7402 cell lines	Melatonin	10 µM 48 h	Apoptosis induction
				Melatonin + Sorafenib	1–100 µM 48 h	Proliferation inhibition
					10 µM 48 h	Apoptosis induction Autophagy blockage
Long et al. 2017 [46]	China	HCC	In vitro Human Bel-7402, SMMC-7721 HCC cell lines Human normal liver L02 cell line	Melatonin	0.2–2 mM 48–72 h	Proliferation inhibition
				Melatonin + Sorafenib	1 mM 48 h 2 weeks	Proliferation inhibition Cell cycle arrest
			In vivo 4-weeks-old female BALB/c nude mice Subcutaneous injection of SMMC-7721 cells (*n* = 4)	Intraperitoneal melatonin	25 mg/kg Daily 18 days	Tumor growth inhibition
				Intraperitoneal melatonin + sorafenib		
Prieto-Domínguez et al. 2017 [4]	Spain	HCC	In vitro Human Hep3B cell line	Melatonin	1 or 2 mM 24 or 48 h	Pro-oxidant activity Proliferation inhibition
				Melatonin + Sorafenib		Proliferation inhibition Blockade of sorafenib-induced mitophagy
Sánchez et al. 2017 [47]	Spain	HCC	In vivo 6-weeks-old male ICR mice Intraperitoneal injection of DEN	Intraperitoneal melatonin	5 or 10 mg/kg Daily 10, 20, 30, 40 weeks	Cell cycle arrest Modulation of sphingolipid metabolism
Wang et al. 2017 [48]	Taiwan	HCC	In vitro Human HepG2 and HuH7 cell lines	Melatonin	1 mM 12, 24, 36, 48, 60 and 72 h	Proliferation inhibition
					1 mM 24, 48 and 72 h	Suppression of cell migration ability
					1 mM 24 h	EMT inhibition
			In vivo 6-to-8-weeks-old male BALB/c nude mice Subcutaneous injection of HuH7 cells (*n* = 10)	Intraperitoneal melatonin	40 mg/kg Five days per week	Tumor growth inhibition EMT suppression
Wongsena et al. 2017 [49]	Thailand	CCA	In vivo 6-to-8-weeks-old male Syrian golden hamsters Oral infection with 50 metacercariae of *Opisthorchis viverrini* and oral administration with DEN (*n* = 7)	Oral melatonin	50 mg/kg Daily 30 days	Immunomodulation
Chen et al. 2018 [50]	Taiwan	HCC	In vitro Human HuH7 and HepG2 cell lines	Melatonin	1 mM 12, 24, 36, 48, 60 and 72 h	Proliferation inhibition
					1 mM 24, 48 and 72 h	Suppression of migration and invasion abilities
				Melatonin + Etoposide	1 mM 12, 24, 36, 48, 60, and 72 h	Proliferation inhibition Apoptosis induction
				Melatonin + Camptothecin	1 mM 24 h	
Chen et al. 2018 [50]	Taiwan	HCC	In vivo 6-weeks-old male BALB/c nude mice Subcutaneous injection of HuH7 cells (*n* = 6)	Intraperitoneal melatonin	40 mg/kg Five days/week 25 days	Tumor growth inhibition Apoptosis induction
				Intraperitoneal melatonin + etoposide		
Colombo et al. 2018 [51]	Brazil	HCC	In vitro Human HepG2 cell line	Melatonin	1 mM 24 h	Increase of NF-κB protein expression
Dauchy et al. 2018 [52]	USA	HCC	In vivo Male Buffalo rats Implantation of Morris 7288CTC hepatomas (control: *n* = 6; experimental: *n* = 9)	Endogenous melatonin	Increase of endogenous melatonin levels	Tumor growth inhibition
Sánchez et al. 2018 [14]	Spain	HCC	In vitro Human Hep3B cell line	Melatonin	0.5 or 1 mM 1 h	Proliferation inhibition Apoptosis induction
				Melatonin + SR9009		Proliferation inhibition
				Melatonin + Bmal1 siRNA	0.5 or 1 mM 24 h	Proliferation inhibition Apoptosis induction
			In vivo 6-weeks-old male ICR mice Intraperitoneal injection of DEN (*n* = 4–8)	Intraperitoneal melatonin	5 or 10 mg/kg Daily 10, 20, 30, 40 weeks	Circadian clock modulation Cell cycle arrest Apoptosis induction
Wang et al. 2018 [53]	Taiwan	HCC	In vitro Human HepG2 and HuH7 cell lines	Melatonin	1 and 2 mM 12, 24, 36, 48, 60 and 72 h	Proliferation inhibition
					1 and 2 mM 24, 48 and 72 h	Suppression of migration and invasion abilities
				Melatonin + let-7i-3p inhibitor	1 and 2 mM 24 and 48 h	Proliferation inhibition Migration and invasion suppression
			In vivo 6–8-weeks-old male BALB/c nude mice Subcutaneous injection of HuH7 cells (*n* = 6)	Intraperitoneal melatonin	40 mg/kg 5 days per week 35 days	Tumor growth inhibition
El-Magd et al. 2019 [54]	Egypt	HCC	In vivo Adult female rats Intraperitoneal injection of DEN and oral administration of 2-AAF at week 2 (*n* = 10)	Intraperitoneal melatonin	20 mg/kg Twice a week 5 weeks	Apoptosis induction Antioxidant activity Reduction of angiogenesis and metastasis
				Intraperitoneal melatonin + MSCs		
				Intraperitoneal injection of MSCs preincubated with melatonin	5 µM 24 h	
Mohamed et al. 2019 [55]	Egypt	HCC	In vivo Adult female rats Intraperitoneal injection of DEN followed by oral administration of 2-AAF at week 2 (*n* = 10)	Intraperitoneal melatonin	20 mg/kg Twice a week 5 weeks	Tumor growth inhibition Apoptosis induction
				Intraperitoneal injection of MSCs preincubated with melatonin 5 µM for 24 h		
Zhang et al. 2019 [56]	China	HCC	In vitro Human HepG2 cell line	Melatonin	3 mM 48 h	Suppression of migration
					1 mM 14 days	Proliferation inhibition
				Melatonin + EGCG	3 mM 48 h	Suppression of migration
					1 mM 14 days	Proliferation inhibition
Zhou et al. 2019 [57]	China	HCC	In vitro Human HepG2, 7721 and HuH7 HCC cell lines Human liver L02 cell line	Melatonin	1–100 µM 48 h	Apoptosis induction
					10 µM 48 h	Autophagy inhibition
				Melatonin + Sorafenib	1–100 µM 48 h	Proliferation inhibition Apoptosis induction
					10 µM 48 h	Autophagy inhibition
Ao et al. 2020 [58]	China	HCC	In vitro Human HepG2 and HuH7 cell lines	Melatonin	2.5 mM 24 h	Apoptosis induction
Mi and Kuang 2020 [59]	China	HCC	In vitro Human HepG2 and Hep3B cell lines	Melatonin	1 or 2 mM 24, 48, 72, 96 h	Proliferation inhibition
					1 or 2 mM 48 h	Cell cycle arrest
				Melatonin + Cisplatin	1 or 2 mM 24 and 48 h	Proliferation inhibition Apoptosis induction
Wang et al. 2020 [60]	China	HCC	In vitro Human SMMC-7721, cell line	Melatonin + Regorafenib	50 µM 24 h	Antioxidant activity Apoptosis induction

2-AAF, 2-acetylaminofluorene; AmB, amphotericin B; BCG, Bacillus Calmette-Guerin; Bmal1, brain and muscle arnt-like protein 1; CCA, cholangiocarcinoma; CDDP, Cis-dichlorodiamineplatinum; DEN, diethylnitrosamine; EGCG, (–)-epigallocatechin-3-gallate; EMT, epithelial-to-mesenchymal transition; ER, endoplasmic reticulum; Exo-con, exosomes from HepG2 cells; Exo-MT, exosomes from melatonin-treated HepG2 cells; HCC, hepatocarcinoma; MF, magnetic field; IL-1β, interleukin 1 beta; IL-2, interleukin-2; MSCs, mesenchymal stem cells; NF-κB, nuclear factor-kappa B; RNAi, interference RNA; siRNA, small interference RNA.

## Supplementary Materials

The following are available online at https://www.mdpi.com/2076-3921/10/1/103/s1, Table S1: PRISMA Checklist.
